# Graphene-Doped Piezoelectric Transducers by Kriging Optimal Model for Detecting Various Types of Laryngeal Movements

**DOI:** 10.3390/mi15101213

**Published:** 2024-09-29

**Authors:** Ming-Chan Lee, Cheng-Tang Pan, Shuo-Yu Juan, Zhi-Hong Wen, Jin-Hao Xu, Uyanahewa Gamage Shashini Janesha, Fan-Min Lin

**Affiliations:** 1 Department of Electrical Engineering, National Kaohsiung University of Science and Technology, Kaohsiung 807, Taiwan; mclee@nkust.edu.tw; 2 Department of Mechanical and Electro-Mechanical Engineering, National Sun Yat-sen University, Kaohsiung 804, Taiwan; pan@mem.nsysu.edu.tw (C.-T.P.); m13175869@gmail.com (S.-Y.J.); 3 Institute of Advanced Semiconductor Packaging and Testing, College of Semiconductor and Advanced Technology Research, National Sun Yat-sen University, Kaohsiung 804, Taiwan; 4 Institute of Precision Medicine, National Sun Yat-sen University, Kaohsiung 804, Taiwan; 5 Taiwan Instrument Research Institute, National Applied Research Laboratories, Hsinchu City 300, Taiwan; 6 Department of Marine Biotechnology and Research, National Sun Yat-sen University, Kaohsiung 804, Taiwan; wzh@mail.nsysu.edu.tw; 7 Division of Pulmonary Medicine, Department of Internal Medicine, Kaohsiung Armed Forces General Hospital, Kaohsiung 802, Taiwan; a1068020014@mail.802.org.tw; 8 Institute of Medical Science and Technology, National Sun Yat-sen University, Kaohsiung 804, Taiwan; 9 Institute of Biomedical Sciences, College of Medicine, National Sun Yat-sen University, Kaohsiung 804, Taiwan; 10 Department of Medical Laboratory Science, Faculty of Allied Health Sciences, University of Ruhuna, Galle 80000, Sri Lanka

**Keywords:** composite piezoelectric fibers, near-field electrospinning, polyvinylidene fluoride, graphene, piezoelectric sensor

## Abstract

This study fabricated piezoelectric fibers of polyvinylidene fluoride (PVDF) with graphene using near-field electrospinning (NFES) technology. A uniform experimental design table ${U^{*}}_{7}\left(7^{4}\right)$ was applied, considering weight percentage (1–13 wt%), the distance between needle and disk collector (2.1–3.9 mm), and applied voltage (14.5–17.5 kV). We optimized the parameters using electrical property measurements and the Kriging response surface method. Adding 13 wt% graphene significantly improved electrical conductivity, increasing from 17.7 µS/cm for pure PVDF to 187.5 µS/cm. The fiber diameter decreased from 21.4 µm in PVDF/1% graphene to 9.1 µm in PVDF/13% graphene. Adding 5 wt% graphene increased the β-phase content by 6.9%, reaching 65.4% compared to pure PVDF fibers. Material characteristics were investigated using scanning electron microscopy (SEM), Fourier-transform infrared spectroscopy (FTIR), X-ray diffraction analysis (XRD), contact angle measurements, and tensile testing. Optimal parameters included 3.47 wt% graphene, yielding 15.82 mV voltage at 5 Hz and 5 N force (2.04 times pure PVDF). Force testing showed a sensitivity (S) of 7.67 log(mV/N). Fibers were attached to electrodes for piezoelectric sensor applications. The results affirmed enhanced electrical conductivity, piezoelectric performance, and mechanical strength. The optimized piezoelectric sensor could be applied to measure physiological signals, such as attaching it to the throat under different conditions to measure the output voltage. The force-to-voltage conversion facilitated subsequent analysis.

## 1. Introduction

With the prevalence of wearable devices, monitoring physiological indicators such as heart rate, blood oxygen saturation, and sleep quality has expanded the smart healthcare market. Advances in biometric technology have elevated sensors to crucial roles in detecting physiological parameters and environmental changes. Through wearable devices, healthcare IoT technology has progressed significantly [1]. Self-powered systems have emerged as the mainstream trend in electronic device development. Traditional batteries, with limitations like limited battery life, low power efficiency, and insufficient energy storage, have prompted researchers to shift toward environmental energy harvesting. This reduces reliance on batteries, extends battery life, and effectively reduces environmental pollution and energy consumption [2]. Among these, pressure sensors are a vital category for detecting pressure magnitude. Common types include capacitive sensors [3,4,5], piezoresistive sensors [6,7], and piezoelectric sensors [8,9].

Piezoelectric materials primarily include piezoelectric ceramics, polymers, biomaterials, and molecular ferroelectrics. Sotov et al. [10] investigated methods for fabricating gradient piezoelectric materials using 3D printing, focusing on barium titanate produced via fused deposition modeling. Qian et al. [11] compared polymer piezoelectric materials and highlighted their potential in biomedical and wearable sensor applications. Zhang et al. [12] reported a method for manufacturing ultra-thin films using submucosa from the small intestine (SIS) structure and demonstrated an SIS biosensor based on this technology. Wang et al. [13] created PVA-GLY nanofiber membranes and developed a self-charging mask with a filtration efficiency exceeding 95%. Zhang et al. [14] found that HOCH_2_(CF_2_)_3_CH_2_OH exhibits good piezoelectric properties and biocompatibility, showing great potential for implantable electromechanical devices. Qi et al. [15] studied TMCM-CdCl_3_ ferroelectric crystals, which maintained good ferroelectric and piezoelectric properties after recrystallization. Polyvinylidene fluoride (PVDF) and ceramic piezoelectric materials offer efficient conversion between electrical and mechanical energy, suitable for filters and sensors. However, ceramics have poor ductility and are prone to damage; while PZT materials exhibit good piezoelectric properties, they contain heavy metals and are harmful to humans. Biological piezoelectric materials have high biocompatibility and flexibility but inferior piezoelectric performance compared to synthetic materials and are costly. Molecular ferroelectrics offer biocompatibility, lightness, and low cost but have inferior performance and stability. PVDF, due to its absence of heavy metals, lightweight nature, low cost, flexibility, high piezoelectric coefficient, and high response frequency, is chosen as the material for this study.

Piezoelectric material-based energy harvesting devices played a crucial role in energy conversion, where, through the piezoelectric effect, mechanical energy could be transformed into electrical energy. These devices detected subtle body movements [16,17,18,19]. PVDF, a polymer piezoelectric material [20,21,22], possesses advantages such as flexibility, light weight, high purity, solvent resistance, and stability under high electric fields. Its molecular structure, composed of repeating units (-CH_2_-CF_2_-), exhibited five crystalline phases (α, β, γ, δ, and ε), with the β phase demonstrating superior piezoelectric, ferroelectric, and pyroelectric properties [23,24]. Enhancement could be achieved through mechanical stretching, high electric field application, and the addition of nanoparticles [25]. Pazhamalai et al. [26] used PVDF in a composite film to enhance energy storage in flexible supercapacitors and create self-powered systems, utilizing its piezoelectric properties for energy harvesting. Li et al. [27] developed a novel piezoelectric polymer material by modifying PVDF with polyvinyl alcohol, significantly improving blood pressure monitoring devices’ electro-mechanical coupling characteristics and accuracy. Graphene [28,29,30], a two-dimensional nanomaterial with carbon atoms bonded in sp^2^ hybridized orbitals, exhibited excellent mechanical properties, high electron transfer capability, and thermal conductivity. It is widely used in biosensors and physical and chemical sensors [31]. Adding graphene to PVDF could modify its properties, forming composite materials with outstanding characteristics. Graphene has excellent properties and is widely applied in various fields. For example, in research related to graphene absorbers, Li et al. [32] proposed a terahertz bandwidth optical device based on graphene. This bandwidth absorber is tunable and can be applied in the field of biomedicine. Shangguan et al. [33] developed an ultra-narrow refractive index sensor with excellent sensing performance, suitable for terahertz photon detection. Jiang et al. [34] proposed an in-fiber photoelectric device by wrapping several layers of graphene around a tilted fiber Bragg grating (TFBG) to perform spectral analysis through real-time monitoring of the photocurrent changes. Shao et al. [35] enhanced the activity of surface-enhanced Raman scattering (SERS) by modulating the energy band of graphene oxide to facilitate charge transfer. Research on graphene-related sensors has been presented. Liu et al. [36] developed a graphene-based flexible sEMG sensor for muscle strength evaluation and hand rehabilitation, combining PDMS substrate for high sensitivity and biocompatibility. Wang et al. [37] used graphene and silver nanoflakes in their pressure sensors to improve flexibility and sensitivity, enabling effective radial pulse monitoring. Hernández-Rivera et al. [38] used graphene in a capacitive humidity sensor with an electrospun PVDF/graphene membrane, enhancing its hydrophobicity and improving its sensitivity and response time for various humidity sensing applications. In addition, measuring the output voltage of piezoelectric materials requires specific techniques and equipment. Šutka et al. [39] discussed the need to consider contact electrification and other factors when measuring the power generation of piezoelectric materials. Chen et al. [40] proposed the need to distinguish between triboelectric and piezoelectric effects when measuring piezoelectric materials. Zhang et al. [41] introduced an active self-assembly strategy to tailor piezoelectric biomaterial thin films. A shaker with a force sensor was used to measure piezoelectric performance. Li et al. [42] proposed electrostatic disk micro-printing technology to improve the piezoelectric strain constant of piezoelectric materials. Cantilever vibration testing was used to measure the output voltage.

Electrospinning [43,44,45,46,47], a technique using a high electric field to manufacture fibers, involves extracting and stretching polymer substances from a liquid into micro or nanoscale fibers. When the electrostatic force exceeds the liquid droplet’s surface tension, the droplet extends into threads, which are collected through a collector. A near-field electrospinning process was proposed by Sun et al. [48], where the needle-to-collector distance was reduced to a range of 500 µm to 3 mm. The shortened distance minimized the applied voltage, improving the severe disturbance during the traditional electrospinning jetting process. This led to successfully fabricating ordered piezoelectric fibers with diameters ranging from 50 to 500 µm. Isaac et al. [49] explored various techniques for producing directional fibers, including using drum collectors to generate high-strength composite fibers. The high-speed rotation of the collector allowed the fibers to stretch and deposit on the collector. Tariq et al. [50] employed the Taguchi design method to set electrospinning parameters, investigating factors such as PVDF concentration, applied voltage, flow rate, and drum speed. Through multiple experiments and modeling, optimal spinning parameters were determined, highlighting the significant impact of PVDF concentration. Wang et al. [51] enhanced the conductivity of PVDF by incorporating multi-walled carbon nanotubes (MWCNT). They observed that higher concentrations of MWCNT significantly increased the β phase, improving the mechanical properties of the fibers. The addition of nanofillers to the polymer, with a minimal needle-to-collector distance, facilitated the optimization method, enhancing the piezoelectric properties of the fibers. Sharma et al. [52] doped MXene (Ti3C2Tx) into PVDF to create nanofibrous scaffolds for ultralow-pressure measurement. This sensor achieved a high sensitivity of 0.51 kPa^−1^ and a minimum detection limit of 1.5 Pa. Additionally, Sharma et al. [53] proposed a piezoresistive sensor based on polyaniline nanospines for monitoring physiological signals. The polyaniline nanospines were fabricated on the surface of nanofibers, enhancing the differences in the contact area, thereby achieving high sensitivity (179.1 kPa^−1^) and a low detection limit (1.2 Pa). Wu et al. [54] fabricated aligned PVDF/CNT nanofibrous membranes through electrospinning, achieving a high piezoelectric sensitivity of 2.26 mV/N under dynamic compression. Selleri et al. [55] developed a self-sensing soft skin based on electrospun PVDF-TrFE nanofibers, achieving a sensitivity of up to 4 mV/N. These studies demonstrate the potential of using piezoelectric fibers for force measurement. Chou et al. [56] proposed a circular-shaped piezoelectric sensor encapsulated with circular electrodes. They combined individual sensors into a sensor array for application in dynamic response measurement.

In this study, graphene was added to PVDF to enhance its conductivity and piezoelectric performance. The near-field electrospinning technique was employed with a disk collector to gather fibers, creating flexible piezoelectric sensors. Table 1 provides a comparison between previous studies and the current research. Through uniform experimental design and kriging modeling, optimal electrospinning parameters for the output voltage of the piezoelectric sensor were determined. Material analyses were conducted to investigate the impact of varying graphene concentrations. Subsequently, the force-to-voltage relationship of the optimized piezoelectric sensor was obtained. The sensor was applied to measure forces on the throat under different conditions.

## 2. Materials and Method

### 2.1. Preparation of PVDF/Graphene Solution

Solution A and B were prepared first by combining 0.9 g of PVDF and 2.5 g of acetone in a scintillation vial to create Solution A. The mixture was stirred at 150 rpm using a magnetic stirrer for 30 min. Subsequently, 0.2 g of anionic phosphate fluorosurfactant (Zonyl UR, Chemours, Wilmington, DE, USA) and 2.5 g of dimethyl sulfoxide (DMSO) were added to another scintillation vial to form Solution B, which was also stirred at 150 rpm using a magnetic stirrer for 30 min. The surfactant used has a low aqueous surface tension. Graphene was incorporated into the pre-prepared Solution B following a uniform experimental design and then placed in an ultrasonic bath for continuous agitation for 20 min. Solution A was added to Solution B, stirring the mixture at 200 rpm for 30 min. After mixing, the solution was allowed to stand for 30 min to reduce the presence of residual bubbles.

### 2.2. Near-Field Electrostatic Spinning Process

**Figure A1:**
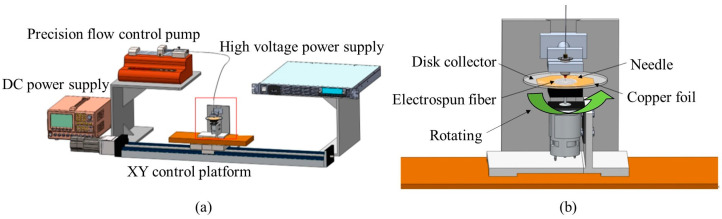
Schematic diagram of near-field electrospinning equipment. (**a**) Overall equipment, (**b**) disk collector and XY platform.

The schematic diagram of the near-field electrospinning setup is illustrated in Figure A1 (Appendix A). The electrospinning solution was initially loaded into a syringe and securely fixed onto the precision flow control pump (NE-1000, New Era Pump Systems, Farmingdale, NY, USA) to regulate the injection rate of the electrospinning solution. A conductive stainless-steel needle connected to the positive high-voltage power supply (AU-60N20-LC, Matsusada Precision, Charlotte, NC, USA) formed the nozzle for near-field electrospinning. A rotating disk collector was employed to gather many spun fibers, with the motor’s direct current power supply (DPS-305CM, HONGSHENG, Shenzhen City, China) controlling the disk’s rotational speed. A glass disk with copper foil underneath was grounded and positioned on an XY control platform for lateral movement. The electrospinning solution formed droplets at the tip of the stainless-steel needle. Under the influence of the electric field, charges gradually accumulate on the droplet’s surface. When the electrostatic force exceeds the droplet’s surface tension, a Taylor cone forms at the needle’s tip, resulting in the ejection of fibers. A high electric field collects the fibers in a specific stretching direction. The experimental electrospinning parameters included the weight percentage of graphene, the distance between the needle and the disk collector, and the applied voltage during electrospinning. These three variables were adjusted using a uniform experimental design method. The XY control platform maintained a fixed movement speed of 8.33 mm/s, and the precision flow control pump maintained a flow rate of 0.15 mL/h.

### 2.3. Sensor Package and Sheet Resistance Measurement

This experiment employed screen printing to fabricate silver electrode films. A 50 µm thick silver electrode was utilized on a flexible polyethylene terephthalate (PET) substrate. The encapsulation part used a flexible plastic material polyethylene (PE) film. The PVDF/graphene piezoelectric fiber was placed on the screen-printed electrode. The copper tape was used to attach the wires to the electrode’s junction, and the piezoelectric fiber was secured on the silver electrode film with a PE film, completing the preparation of the piezoelectric sensor.

The four-point probe method (Kelvin technique) is commonly employed to measure the resistance of thin films. This method eliminates the influence of contact resistance, providing relatively accurate resistance values. The sheet resistance, an essential characteristic for evaluating electrode conductivity, is utilized to assess the resistance value on the electrode surface. This helps confirm the impact of non-uniform thickness or material distribution in different regions and evaluate the uniformity of the silver ink-printed electrodes.

### 2.4. Electrical Measurements

**Figure A2:**
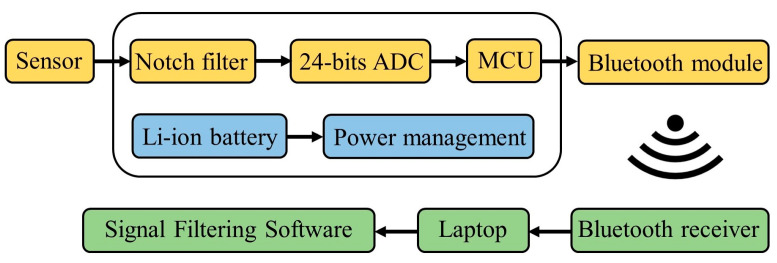
The circuit flowchart schematic of the signal filtering processor illustrates the signal processing and transmission of the output voltage from the piezoelectric sensor.

To assess the performance of the piezoelectric sensor, this study utilized a signal filtering processor provided by the Instrument Technology Research Center, Taiwan. The circuit structure and flowchart of the signal-filtering processor are illustrated in Figure A2 (Appendix A). This processor boasts high-precision detection capabilities and an active filtering function. This circuit was powered by a lithium-ion battery for signal processing and wireless transmission. Although piezoelectric fibers could generate energy from body movements, they could not power the entire circuit. The lithium battery has a specification of 3.7 V and 500 mAh, and the processor consumes approximately 5 mW of power. If continuous measurements are taken, the theoretical usage time is about 370 h. After connecting the piezoelectric sensor to the signal filtering processor, the signal undergoes notch filtering to eliminate the 60 Hz frequency signal, enhancing the clarity of the voltage signal. Subsequently, the analog signal is converted to a 24-bit high-resolution digital signal using an analog-to-digital converter (ADC). Further processing is carried out by the microcontroller unit (MCU). The processed signal is transmitted to a laptop via a Bluetooth module, and signal-filtering software is utilized for recording and analysis. The software employs fast Fourier transform (FFT) to transform the time waveform of the signal into a spectrum, facilitating the identification of frequencies that impact the piezoelectric sensor.

**Figure A3:**
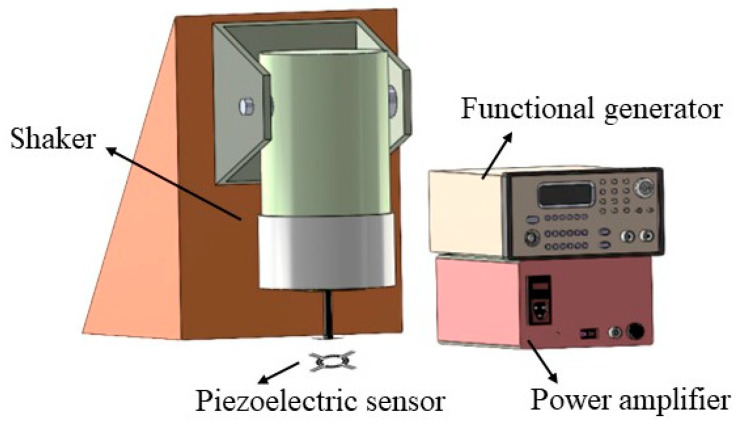
Schematic diagram of electrical measurement equipment for tapping tests to measure the output voltage of piezoelectric fibers.

Next, the piezoelectric sensor was connected to the signal processing unit for impact testing to understand its electrical performance. The sensor was tapped using a shaker to generate voltage output. Initially, a signal with a frequency of 5 Hz was generated using a function generator (Agilent 33220A, Keysight Technologies, Santa Rosa, CA, USA). The force of the tapping was adjusted by manipulating the knob of the power amplifier (SignalForce 100W, Data Physics, Riverside, CA, USA). A shaker (Dataphysics V20, Data Physics, Riverside, CA, USA) was connected, and its tapping head was aligned with the piezoelectric sensor. The setup is illustrated in Figure A3 (Appendix A). A force sensor (Model 208C02 ICP^®^ Force Sensor, PCB Piezotronics, Inc., Depew, NY, USA) was positioned below to measure the tapping force. The knob of the power amplifier was adjusted to achieve a tapping force of 5 N. Under these tapping conditions, the piezoelectric performance of sensors produced with different parameters using a uniform experimental design was measured.

### 2.5. Uniform Experimental Design and Kriging Response Surface Method

The uniform experimental design is a tool for experimental design suitable for situations with multiple factors and levels. Its characteristics include fewer experiments and a uniform distribution of test points. The Kriging response surface method is a spatial data interpolation method used for modeling and optimizing multivariate functions. It aims to determine the optimal combination of multiple factors for a specific response. Constructing a suitable Kriging response surface model can optimize and analyze the surface.

The symbols used in the uniform experimental design are denoted as U_n_(q^s^), where U represents the uniform design, n indicates the number of experiments, q signifies the number of levels for each factor, and s denotes the number of factors. The uniform design also uses the parameter D to represent the distribution of actual sample points, reflecting the degree of uniformity in the experimental design. A smaller D value indicates a more uniform distribution of sample points. This experiment utilized the specifications of the U*7 (74) uniform experimental design method. It consists of four columns, and three influencing factors were placed in columns 2, 3, and 4 for experimentation. The influencing factors include the graphene weight percentage, the distance between the needle and the disk collector, and the applied voltage in the electrospinning process. Each factor has specified upper and lower limits.

A uniform experimental design table was created for conducting experiments. Table 2 and Table 3 present the upper and lower limits of the influencing factors and the uniform experimental design table. A range of 1–13 wt% graphene weight percentage was chosen. Although higher concentrations result in higher conductivity, they also increase the viscosity of the graphene solutions, making the electrospinning process more challenging. Consequently, higher electric fields need to be applied. A uniform design table was used to address this issue. Adjustments were made in the distance between the needle and the disk collector in the uniform table and the range of applied voltages during electrospinning. The corresponding electric field is calculated as the applied voltage divided by the distance between the needle and the collector. This adjustment ensures that higher concentrations of graphene are matched with higher electric fields, enabling a more stable electrospinning process. This prevents the formation of spherical aggregates on the needle due to high viscosity, which could otherwise lead to the inability to form fibers as they drop down during electrospinning.

### 2.6. Scanning Electron Microscope, SEM

Due to the fiber dimensions reaching the micron scale, observing subtle structural changes using an optical microscope is challenging. SEM offers higher magnification, ranging from thousands to hundreds of thousands of times, enabling clear visualization of surface features and delicate structures. Therefore, SEM was utilized to examine the diameter and surface morphology of PVDF/graphene composite fibers.

### 2.7. Electric Conductivity

Electrical conductivity is an assessment of a material’s ability to conduct electricity. It indicates the mobility of electrons or ions in the material under an applied electric field and indicates the material’s efficiency in transmitting electric current. The objective is to observe whether adding graphene effectively enhances the electrical conductivity of the PVDF/graphene electrospinning solution.

### 2.8. Fourier-Transform Infrared Spectroscopy, FTIR

The FTIR technique is employed to characterize and analyze the crystalline phases of PVDF material. It is used to observe whether adding graphene increases the presence of PVDF β-phase functionalities. FTIR analysis also helps determine the relative content and distribution of various crystalline phases in the material by analyzing the positions and intensities of different absorption peaks. It also assists in identifying the presence of other functional groups within the molecules.

### 2.9. X-ray Diffraction Analysis, XRD

XRD analysis was employed to identify the crystal structures of PVDF and graphene to investigate the impact of adding different concentrations of graphene on the β-phase crystallinity strength. Before measurement, approximately 1 × 1 cm fibers were cut, their surfaces were flattened, and they were adhered to glass slides. The materials’ crystalline structures were analyzed by comparing the XRD peak angles corresponding to graphene and PVDF crystal structures.

### 2.10. Contact Angle Measurement

To compare the influence of different concentrations of graphene on the hydrophilicity of composite fibers, the contact angle (θ) was used as an indicator for evaluation. The electrospun fibers were placed on a substrate, and a droplet of 2 µL distilled water was dispensed onto the fiber surface.

### 2.11. Tensile Testing

Tensile testing was employed to assess whether the addition of graphene affects the crystalline structure of PVDF and its impact on mechanical strength. The test samples were cut to a width of 10 mm and a length of 40 mm. The specimens were securely clamped in the grips, and the effective gauge length was 20 mm. Tensile tests were conducted at a rate of 5 mm/min, subjecting the samples to continuous tensile loading until reaching their ultimate strength, resulting in the fracture of the samples.

## 3. Results and Discussion

### 3.1. Sheet Resistance Values of Electrode Design

This experiment utilized silver electrode films with a thickness of 50 µm to fabricate piezoelectric sensors. Due to the small thickness, sheet resistance was employed as an indicator to assess the conductivity of the electrode films. The sheet resistance values were obtained at ten randomly selected points using a four-point probe to measure the uniformity of the silver electrode films produced by screen printing. The individual sheet resistance values are presented in Table 4. These ten points’ average sheet resistance value was 54.82 mΩ/square, with a standard deviation of 0.9978 mΩ/square. It can be observed that the sheet resistance values at different points of the silver electrode film exhibit minimal variations, indicating excellent uniformity.

### 3.2. Conductivity Measurement of the Solution

**Figure A4:**
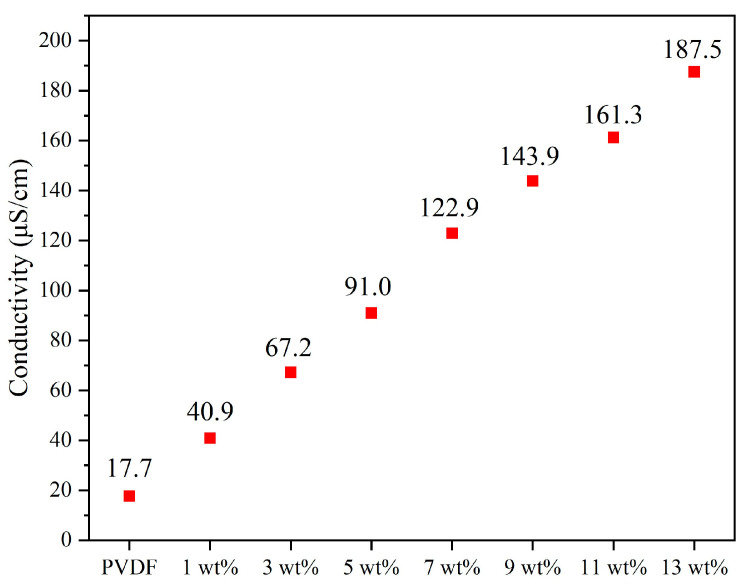
The relationship between different concentrations of graphene solutions and electrical conductivity demonstrates that higher graphene concentrations result in higher electrical conductivity.

In this experiment, the electrical conductivity of the electrospinning solution with different weight percentages of graphene was measured, as shown in Figure A4 (Appendix A). The results indicate a significant increase in electrical conductivity as the concentration of graphene increases. The electrical conductivity of pure PVDF is 17.7 µS/cm. When 13 wt% graphene is added, the conductivity of the PVDF electrospinning solution reaches 187.5 µS/cm. This demonstrates a progressive improvement in conductivity with increasing graphene concentration.

### 3.3. FTIR Analysis of the Crystal Structure of Piezoelectric Fibers

**Figure 1:**
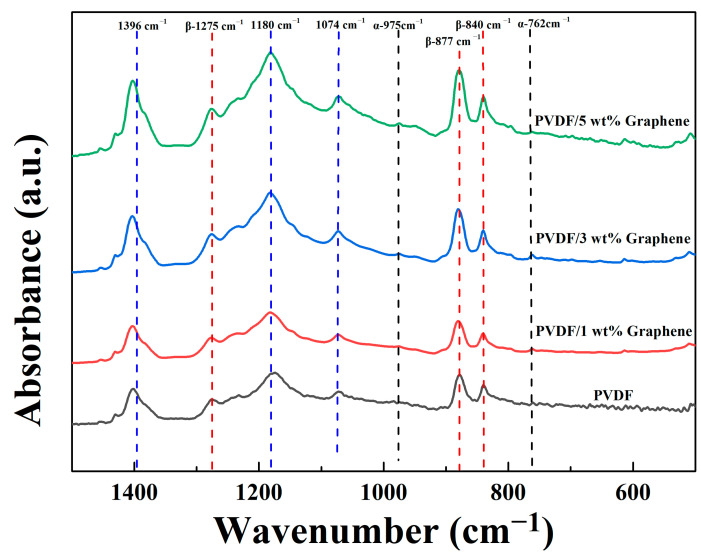
The FTIR analysis results of the piezoelectric fibers in the wavenumber range of 500 cm^−1^ to 1500 cm^−1^ exhibit absorption peaks for pure PVDF and 1 wt%, 3 wt%, and 5% wt% PVDF/graphene compositions.

Figure 1 displays the FTIR spectra of fibers with different graphene concentrations in the wavenumber range of 500 cm^−1^ to 1500 cm^−1^. As shown in the figure, both PVDF fibers and PVDF/graphene composite fibers exhibit absorption peaks at 1180 cm^−1^, 1074 cm^−1^, and 1396 cm^−1^, indicating similar structural features attributed to CF_2_ symmetric stretching, C-C asymmetric stretching, and CH_2_ rocking vibrations, respectively. The α-phase absorption peaks are observed at 762 cm^−1^ (CF_2_ bending and rocking vibrations) and 975 cm^−1^ (CH_2_ torsional vibration). The β-phase absorption peaks appear at 840 cm^−1^ (CH_2_ wagging and CF_2_ asymmetric stretching), 877 cm^−1^ (C-C asymmetric stretching), and 1275 cm^−1^ (CF_2_ symmetric stretching). The β-phase content percentage of the fiber samples is calculated using Equation (1) [57]:

(1) $$F\left(\beta \right)=\frac{A_{\beta }}{(\frac{K_{\beta }}{K_{\alpha }}{)A}_{\alpha }+A_{\beta }}$$

where A_α_ and A_β_ represent the absorbance values of the peaks at 762 cm^−1^ and 840 cm^−1^, respectively. The absorption coefficients are given as K_α_ = 6.1 × 10^4^ cm^2^ mol^−1^ and K_β_ = 7.7 × 10^4^ cm^2^ mol^−1^. The β-phase content percentages for pure PVDF and fibers with 1 wt%, 3 wt%, and 5 wt% graphene are 58.5%, 62.1%, 65.0%, and 65.4%, respectively. Adding 5 wt% graphene increases the β-phase content by 6.9% compared to pure PVDF fibers. These results indicate that the introduction of graphene promotes the crystalline transformation of PVDF, inducing the formation of a higher percentage of β-phase structures with piezoelectric properties.

### 3.4. XRD Analysis of the Crystal Structure of Piezoelectric Fibers

**Figure 2:**
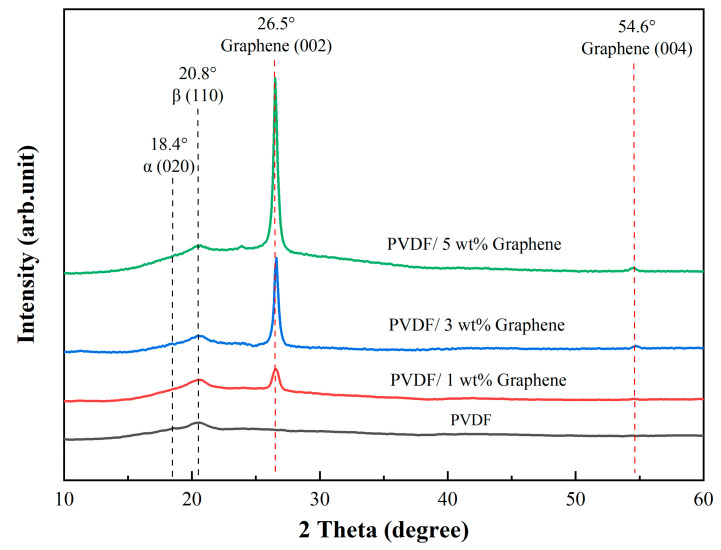
The XRD spectrum of piezoelectric fibers showcases characteristic peaks for pure PVDF and 1 wt%, 3 wt%, and 5 wt% PVDF/graphene compositions.

Figure 2 illustrates the X-ray diffraction patterns of the prepared fibers in this study. The characteristic peak of the PVDF fibers is approximately 18.4°, corresponding to (020). This peak transforms into a broadened peak, suggesting a partial transformation from the α-phase to the β-phase in the PVDF fibers. Additionally, the diffraction peak at 2θ angles mainly occurs at 20.8°, corresponding to the (110) crystal planes, becoming the prominent characteristic peak, indicating the presence of the β-phase in the material [58]. For fibers with the addition of 5 wt% graphene, two characteristic peaks are observed at around 26.5° and 54.6°, corresponding to the reflections of the graphene crystal planes (002) and (004), respectively [59]. These two characteristic peaks are commonly observed in the X-ray diffraction pattern of graphene. The PVDF solution with the addition of graphene exhibits a significant increase in the peak intensity associated with the β-phase compared to the original PVDF solution without graphene. The addition of 3 wt% and 5 wt% graphene shows the presence of a characteristic peak at 54.6° (004), while 1 wt% graphene does not exhibit a characteristic peak at this position. This may be attributed to the lower concentration of 1 wt% graphene, resulting in insufficient crystal content, and the characteristic peak may be very weak or challenging to detect. However, adding a low concentration of 1 wt% graphene enhances the β-phase. With the increase in graphene concentration, the characteristic peak at 26.5° (002) becomes sharper, indicating an improvement in the crystallinity of the sample.

### 3.5. Measurement of Hydrophobicity of Piezoelectric Fibers

**Figure 3:**
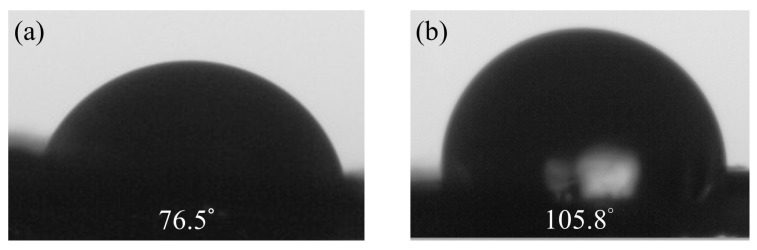
The water contact angle results for fibers. (**a**) Pure PVDF, (**b**) 5 wt% graphene.

Hydrophobicity measurements primarily aim to assess the changes in the surface properties of materials after adding graphene to pure PVDF. By measuring the water contact angle, the hydrophilic or hydrophobic properties of the materials can be understood. This information directly impacts the stability and performance of sensors and contributes to the design and optimization of sensors. Graphene possesses a high surface area and excellent hydrophobicity, making it a common additive in composite materials. The water contact angle of PVDF films prepared by electrospinning without graphene is 76.5°. However, with 5 wt% graphene, the water contact angles increase to 105.8°, showing an upward trend, as illustrated in Figure 3. The introduction of graphene promotes the formation of more β-crystal phases, possibly due to graphene restricting the molecular chain movement of PVDF, leading to the generation of more β-crystal phases during the electrospinning process. The results indicate that the nanostructure of PVDF/graphene composite fibers increases their surface roughness, thereby enhancing the hydrophobicity of the films.

### 3.6. Mechanical Properties of Piezoelectric Fibers

**Figure 4:**
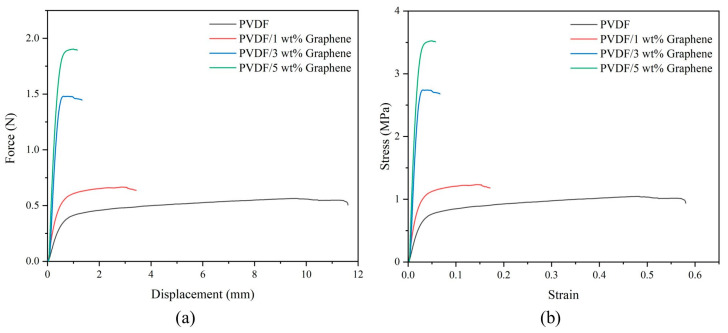
Tensile test of pure PVDF and 1 wt%, 3 wt%, and 5 wt% PVDF/graphene fibers. (**a**) Force–displacement curve, (**b**) stress–strain curve.

This study used an AGS-50KNXD universal testing machine (Shimadzu, Kyoto, Japan) for tensile tests. The fibers were cut into samples with a width of 10 mm and a length of 40 mm. The samples were clamped in the fixture, with an actual gauge length of 20 mm, and tensile tests were conducted at a rate of 5 mm/min. The samples were subjected to tensile loading until they reached their ultimate strength and fractured. The use of piezoelectric fibers depends on their ability to withstand specific force loads. Therefore, this experiment investigates the impact of graphene addition on the mechanical properties of PVDF fibers. The effect of graphene on the mechanical performance of PVDF is shown in Figure 4. The end of the curves indicates fiber rupture. From the figure, it can be observed that before the addition of graphene, PVDF exhibits a tensile maximum force of 0.565 N (tensile strength of 1.04 MPa) and a displacement at fracture of 11.61 mm (fracture strain of 0.581), indicating lower tensile strength and higher flexibility, characteristic of a pliable material. As the graphene content increases, the tensile maximum force increases, and the displacement at fracture decreases. At 5 wt% graphene, these values are 1.909 N (tensile strength of 3.52 MPa) and 1.152 mm (fracture strain of 0.058), respectively, demonstrating the characteristics of a brittle material. Young’s modulus is calculated based on the slope of the elastic region of the stress–strain curve. For the PVDF fibers, Young’s modulus increases significantly from 25.65 MPa in the pure form to 135.92 MPa with the addition of 5 wt% graphene. The high surface area of graphene enhances interfacial interactions, inducing intercalation between PVDF molecular chains and graphene layers in the crystalline region of the composite fibers. This transformation changes PVDF from a pliable material to a brittle one, increasing PVDF strength.

### 3.7. Piezoelectric Fiber Tapping Output Voltage

**Figure 5:**
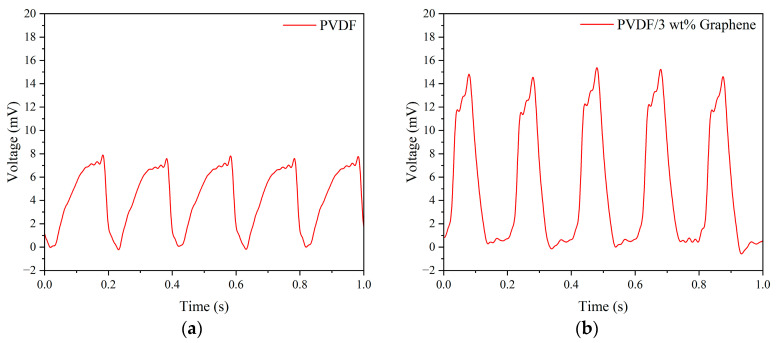
The output voltage of (**a**) pure PVDF and (**b**) 3 wt% graphene piezoelectric fibers under 5 Hz tapping frequency and 5 N force.

The electrical properties of piezoelectric fibers made with different concentrations of graphene were analyzed. The fibers were subjected to vertical tapping using a fixed frequency of 5 Hz and a force of 5 N applied by a tapping device. Figure 5 illustrates the output voltage for pure PVDF and PVDF fibers with 3 wt% graphene added under these tapping conditions. The average maximum output voltage for pure PVDF fibers was 7.74 mV. Among all concentrations of graphene, the composite fibers with 3 wt% graphene exhibited the highest output voltage, with an average maximum output voltage of 14.93 mV, approximately 1.93 times that of pure PVDF fibers. The results indicate superior performance for fibers with added graphene compared to pure PVDF fibers.

**Figure 6:**
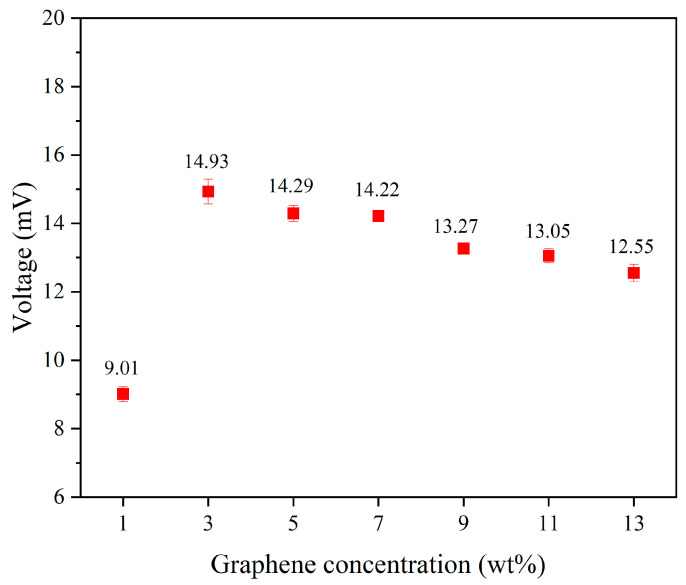
The average output voltage values of piezoelectric fibers with graphene concentrations range from 1 to 13 wt%.

Additionally, tapping tests were conducted on the uniform experimental design table to measure the output voltage of 1–13 wt% graphene under these tapping conditions. Among them, the composite fiber containing 3 wt% graphene exhibited the highest output voltage, with an average peak output voltage of 14.93 mV. Figure 6 presents the output voltage values of piezoelectric fibers with different concentrations added. From the results, it can be observed that the output voltage of high-concentration graphene fibers showed a decreasing trend. This is due to the tendency of high-concentration graphene to aggregate, preventing complete and uniform mixing with PVDF. Moreover, the electrospinning process becomes more challenging due to the high viscosity of the solution, leading to a decrease in the power generation capability of the fibers. We noted that increased graphene concentrations lead to higher solution viscosity, which presents challenges during the electrospinning process. To address this, we implemented a uniform design to optimize the parameters, facilitating the production of fibers more effectively.

### 3.8. Kriging Method Result

**Figure 7:**
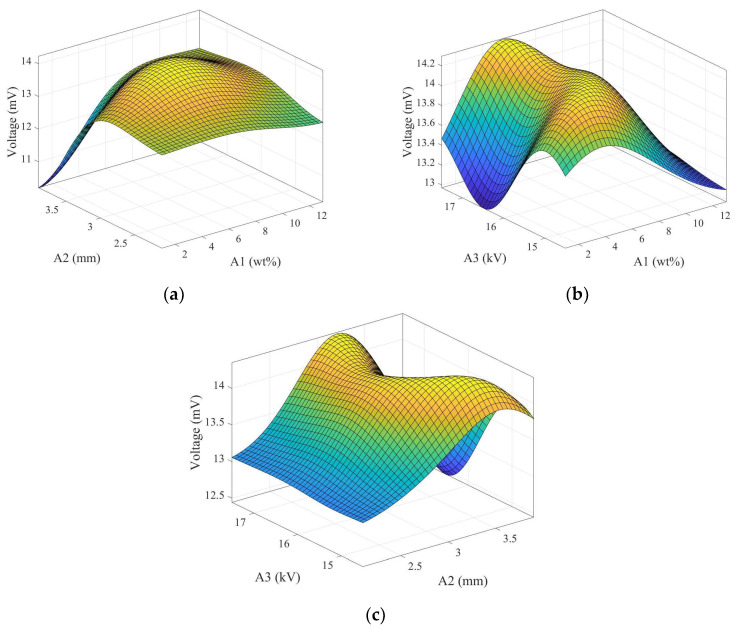
Kriging response surfaces for two factors from the uniform experimental design table. (**a**) Graphene weight percentage (wt%) and the distance between the needle and the disk collecting device (mm), (**b**) graphene weight percentage (wt%) and applied voltage (kV), and (**c**) distance between the needle and the disk collecting device (mm) and applied voltage (kV).

To understand the relationship between the controlled factors in the uniform experimental design and the voltage output performance of electrospun fibers in this study, the Kriging response surface method was employed, denoted by the code numbers shown in Table 5. Initially, a set of experimental parameters was designed using the uniform experimental design method, with the normalized values of the three controlled factors (ranging from 0 to 1) as input for the model and the output voltage from the experimental tapping test. The relationship between the experimental control factors was obtained through this response surface model, as illustrated in Figure 7. A1 represents the weight percentage of graphene, A2 is the distance between the needle and the disk-collecting device, and A3 is the applied voltage. Each plot shows the interaction between two controlled factors and the voltage output of electrospun fibers. The highest point on each surface plot represents the optimal parameters for the piezoelectric effect of the electrospun fibers.

**Figure 8:**
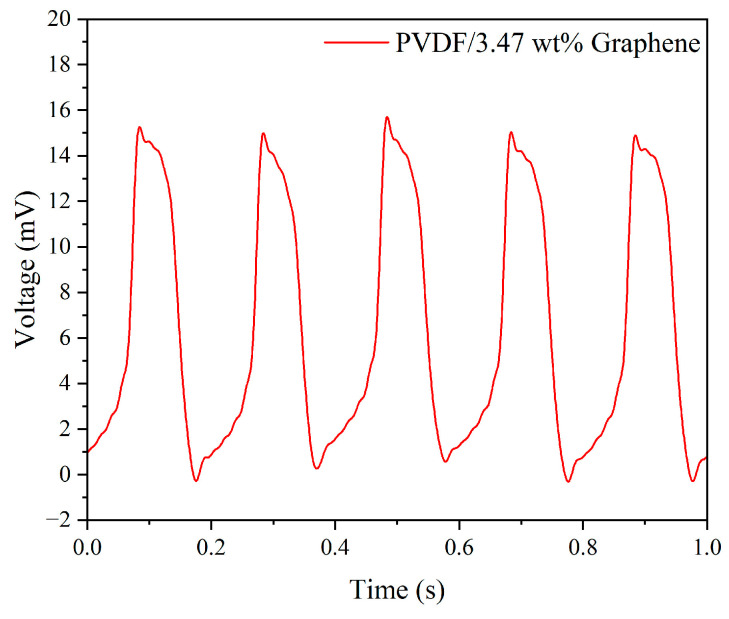
The output voltage of the PVDF/3.47 wt% graphene piezoelectric sensor under 5 Hz tapping frequency and 5 N force.

The experimental results of the uniform design method, combined with the Kriging response surface model, yielded the optimal experimental control factor parameters for the best piezoelectric performance of electrospun fibers, as shown in Table 5. Subsequently, using the obtained optimal parameter combination, fibers were manufactured and encapsulated into a piezoelectric sensor for impact testing. Figure 8 displays the output voltage of the optimized piezoelectric sensor under 5 Hz frequency and 5 N force conditions, with a voltage value of 15.82 mV, representing a 2.04 times increase compared to pure PVDF.

**Figure 9:**
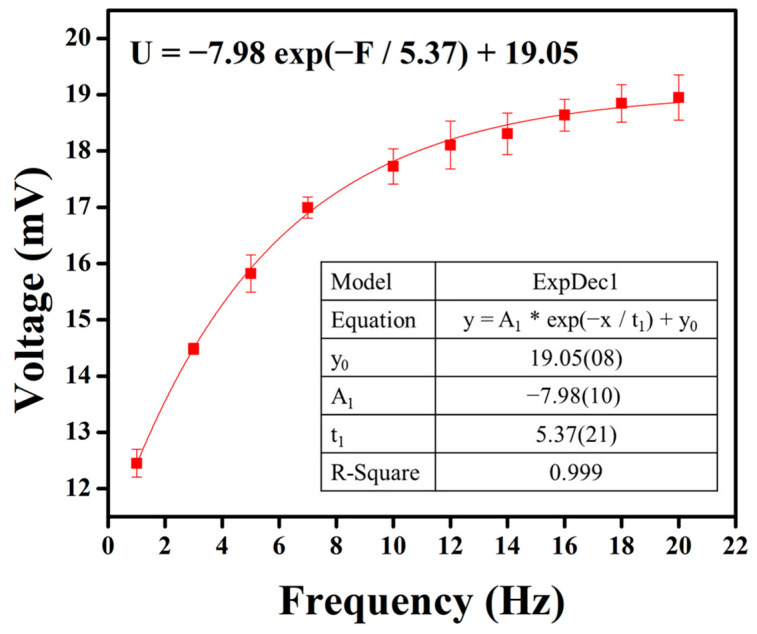
The results of the PVDF/3.47 wt% graphene piezoelectric sensor for the output voltage across the frequency range of 1–20 Hz. Higher frequencies correspond to higher output voltages. As the frequency increases further, the voltage output approaches a limit.

Subsequently, frequency tests were conducted on the optimized parameters of the piezoelectric sensor, tapping at frequencies ranging from 1 to 20 Hz under a 5 N force. The relationship between each frequency and the voltage output of the piezoelectric sensor is illustrated in Figure 9. The experimental results show that as the tapping frequency increases, there is a trend of elevated output voltage. This suggests that the higher frequency of impacts applied to the piezoelectric material per second may lead to more frequent deformation of the crystal structure, resulting in increased charge separation. The frequency–voltage relationship was modeled using curve fitting, and the relationship is described by the equation U = −7.98 exp(−F/5.37) + 19.05. As the frequency continues to rise, the voltage output approaches a limit of 19.05 mV.

**Figure 10:**
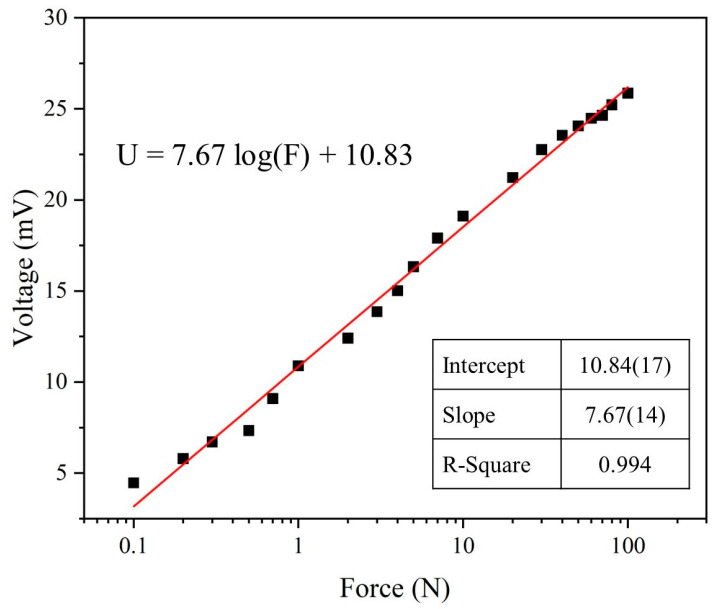
The force-to-voltage graphs of the PVDF/3.47 wt% graphene piezoelectric sensor demonstrate the relationship between force and voltage conversion.

Finally, force tests were conducted on the optimized parameters of the piezoelectric sensor, subjecting it to impacts ranging from 0.1 N to 100 N at a frequency of 5 Hz. The relationship between each force level and the sensor’s output voltage is illustrated in Figure 10. The force-to-voltage conversion is represented using a logarithmic scale for the force. The relationship is described by the equation U = 7.67 log(F) + 10.83, where U is the voltage in millivolts (mV) and F is the force in newtons (N). The sensitivity (S) of the piezoelectric sensor is 7.67 log(mV/N), with a determination coefficient (R^2^) of 0.994. The force-to-voltage relationship graph provides a means to assess the magnitude of the force acting on the piezoelectric sensor.

### 3.9. Surface Morphology of Fibers

**Figure 11:**
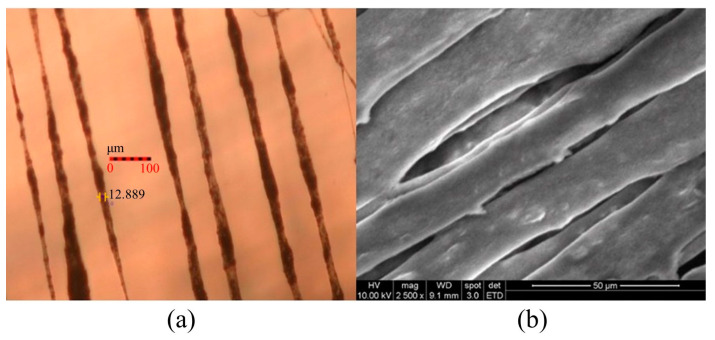
Observing the surface morphology of PVDF/3.47 wt% graphene piezoelectric fiber through (**a**) optical microscope analysis at 10× magnification and (**b**) SEM analysis at 2500× magnification.

With the increased electrostatic field, the Taylor cone formed by the PVDF solution enlarges, resulting in a higher jetting rate, increased stretching force, and reduced fiber diameter. Graphene enhances conductivity, making the solution easier to eject, reducing the resistance during the electrospinning process, expanding the ejection rate, and decreasing the fiber diameter. Near-field electrospinning technology allows for the ordered arrangement of PVDF solution, and compared to traditional electrospinning processes, the prepared piezoelectric fibers exhibit directional alignment. Figure 11, respectively, depicts the morphology of PVDF/3.47 wt% graphene fibers under optical microscopy and SEM. In the uniform design table, the electric field magnitudes for the PVDF/1% graphene and PVDF/13% graphene piezoelectric fibers are 4.23 × 10^6^ V/m and 7.38 × 10^6^ V/m, respectively. The corresponding average fiber diameters are 21.4 and 9.1 µm. The corresponding average fiber diameters are 28.2 and 13.7 µm. With the increase in graphene concentration, conductivity improves, electric field strength increases, and the fiber diameter shows a decreasing trend. An observation of the fiber surface structure after adding 3.47 wt% graphene reveals increased roughness, possibly due to graphene aggregation, leading to the formation of tiny pores.

### 3.10. Application of Physiological Signals in Throat Region

**Figure A5:**
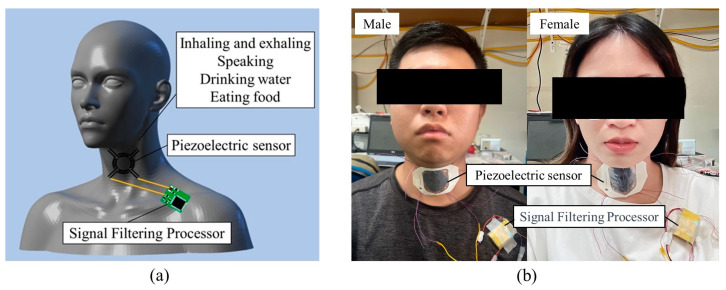
Application for the throat region. (**a**) Throat strength signal measurement schematic, (**b**) the testing process for male and female participants.

The piezoelectric sensor-optimized parameters were applied to measure physiological signals in the human body. The sensor was attached to the throat using a transparent film dressing (1624PP-6, 3M Tegaderm, St. Paul, MN, USA) for force measurement and analysis and connected to a signal-filtering processor positioned below the clavicle. This dressing offered stable adhesion and hypoallergenic properties, securely adhering to moderately moist skin while being breathable and waterproof, effectively blocking dust and enhancing comfort. Male and female participants underwent testing in four different conditions: inhalation/exhalation (three complete breathing cycles within 10 s, each peak from the beginning of inhalation to the end of exhalation), speaking (one word per second), drinking water (50 mL), and eating food (a small piece of bread). The force exerted during different throat movements was observed, allowing for subsequent analysis. Figure A5a,b (Appendix A) show the process of using the piezoelectric sensor and signal filtering processor under the four conditions and the physical measurements for male and female participants, yielding voltage signals under different test conditions, respectively.

**Figure 12:**
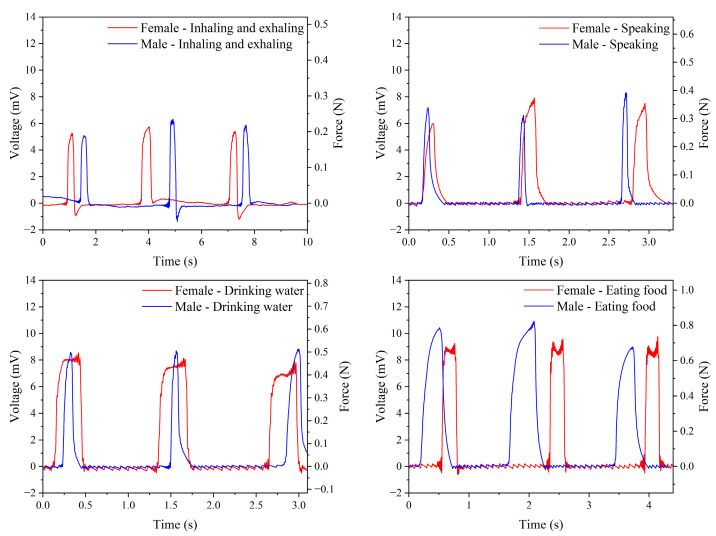
Measurements of the output voltage and force from piezoelectric sensors attached to the throats of male and female participants under four different conditions.

Based on the previously completed force conversion to voltage, Figure 12 presents the output voltage and corresponding force for male and female participants under four conditions. For female participants, the average output voltages during inhaling/exhaling, speaking, drinking water, and eating are 5.47 mV, 7.14 mV, 8.21 mV, and 9.55 mV, respectively. The corresponding forces are 0.200 N, 0.330 N, 0.455 N, and 0.680 N. For male participants, the average output voltages under the same conditions are 5.76 mV, 7.39 mV, 8.72 mV, and 10.12 mV, with corresponding forces of 0.218 N, 0.356 N, 0.530 N, and 0.806 N. Table 6 provides a comparison of all measured data, indicating that male output forces are generally higher than female forces under all conditions. The four conditions, ranked by descending output force, are eating food, drinking water, speaking, and inhaling/exhaling. This is attributed to the more significant fluctuations in the throat while eating food than while inhaling/exhaling, which involves minimal volatility. These test results demonstrate the feasibility of converting voltage signals to force signals using piezoelectric sensors, showcasing potential applications in health monitoring systems. The precision of piezoelectric sensors extends their utility beyond medical settings to possible applications in personal health management, including monitoring swallowing and assessing vocal health. These non-invasive capabilities provide real-time insights crucial for enhancing personal health.

## 4. Conclusions

This study utilized near-field electrospinning to fabricate PVDF/graphene composite piezoelectric fibers. Optimal electrospinning parameters for achieving the highest output voltage were obtained through a uniform design method. Under 5 Hz frequency and 5 N force tapping conditions, the output voltage reached 15.82 mV, 2.04 times that of pure PVDF. The relationship between force and output voltage was investigated under different tapping forces. The addition of graphene enhanced the electrical conductivity. SEM observations of pure PVDF and graphene-added fibers revealed that higher electric fields and conductivity reduced fiber diameter. FTIR and XRD analyses further confirmed crystalline transformations and the formation of the β-phase. The β-phase content in fibers with 5 wt% graphene was 65.4%, representing a 6.9% increase compared to pure PVDF fibers. Water contact angle measurements demonstrated enhanced hydrophobicity with graphene addition. Mechanical testing revealed a transition from ductile to brittle behavior with the addition of graphene, leading to increased tensile strength. Finally, the optimized piezoelectric sensor was applied to measure throat forces. For female participants, the average output voltage and force ranged from 5.47 mV (0.200 N) to 9.55 mV (0.680 N), while for male participants, the range was 5.76 mV (0.218 N) to 10.12 mV (0.806 N). The results indicated higher output forces in males, with the maximum force observed during eating. This study developed a piezoelectric sensor, demonstrating promising applications in physiological measurements.

## Figures and Tables

**Table 1:** Difference between previous studies and the current study.

References	Method	Material	Objective
[38]	Electrospinning Plate collector	PVDF/Graphene	Fabricating capacitive humidity sensors.
[50]	Electrospinning Drum collector	PVDF/DMF	Using the Taguchi design method to enhance the β-phase of PVDF.
[51]	Electrospinning Drum collector	PVDF/MWCNT	We are improving the mechanical and electrical properties of piezoelectric fibers.
[54]	Electrospinning Drum collector	PVDF/CNT	Improving the β-phase and electrical properties of piezoelectric fibers.
[55]	Electrospinning Drum collector	PVDF-TrFE	Fabricating self-sensing soft skin.
This work	Electrospinning Disk collector	PVDF/Graphene	Using the uniform design method to fabricate optimal sensors for throat applications.

**Table 2:** The upper and lower limits of the influencing factors for the uniform experimental design table include graphene weight percentage, the distance between the needle and the disk collector, and applied voltage.

Factor	Graphene Weight Percentage (wt%)	Distance between the Needle and the Disk Collector (mm)	Applied Voltage (kV)
Minimum	1	2.1	14.5
Maximum	13	3.9	17.5

**Table 3:** The influencing factors, including graphene weight percentage, the distance between the needle and the disk collector, and applied voltage, along with their corresponding electric fields, were incorporated into the U*7 (74) uniform experimental design table.

	Graphene Weight Percentage (wt%)	Distance between the Needle and the Disk Collector (mm)	Applied Voltage (kV)/Electric Field Intensity (kV/mm)
1st test	5	3.3	17.5/5.30
2nd test	11	2.4	17.0/7.08
3rd test	1	3.9	16.5/4.23
4th test	7	3.0	16.0/5.33
5th test	13	2.1	15.5/7.38
6th test	3	3.6	15.0/4.17
7th test	9	2.7	14.5/5.37

**Table 4:** The sheet resistance at ten randomly selected points on the silver electrode demonstrates its good uniformity.

The Sheet Resistance at Ten Randomly Selected Points										
Measurement Point Number	1	2	3	4	5	6	7	8	9	10
Sheet resistance (mΩ/sq)	53.2	54.8	54.8	53.7	54.5	54.8	54.8	57.2	55.3	55.1

**Table 5:** The optimized parameters were obtained through the Kriging response surface using the parameters and output voltage values from the uniform experimental design.

Factor	Graphene Weight Percentage (wt%)	Distance between the Needle and the Disk Collecting Device (mm)	Applied Voltage (kV)/Electric Field Intensity (kV/mm)
Optimized parameters	3.47	3.53	14.87/4.21

**Table 6:** The output voltage and force data of male and female participants, under four different conditions, show minimal force during inhaling/exhaling and maximal force while eating food.

Gender	Inhaling/Exhaling		Speaking		Drinking Water		Eating Food	
	Voltage (mV)	Force (N)	Voltage (mV)	Force (N)	Voltage (mV)	Force (N)	Voltage (mV)	Force (N)
Female	5.47	0.200	7.14	0.330	8.21	0.455	9.55	0.680
Male	5.76	0.218	7.39	0.356	8.72	0.530	10.12	0.806

## Data Availability

The raw data supporting the conclusions of this article will be made available by the authors upon request.

## Appendix A

Figure A1 shows the near-field electrospinning setup, including a precision flow control pump, a high-voltage power supply, and a rotating disk collector. A glass disk with copper foil, grounded and positioned on an XY control platform, collected the electrospun fibers. Figure A2 illustrates the circuit structure and flowchart of the signal-filtering processor used in this study. The processor, powered by a lithium-ion battery, was employed for high-precision detection and the active filtering of signals from the piezoelectric sensor. The processed signals were then transmitted to a laptop for analysis. Figure A3 illustrates the setup used for tapping testing of the piezoelectric sensor. The sensor was tapped using a shaker connected to a function generator and a power amplifier. A force sensor was positioned below the tapping head to measure the force applied during the testing. Figure A4 shows the measured electrical conductivity of the electrospinning solution with varying weight percentages of graphene. The results indicated a significant increase in conductivity as the graphene concentration increased. Figure A5 shows how the piezoelectric sensor, attached to the throat with a transparent film dressing, recorded voltage signals during inhalation/exhalation cycles, speaking, drinking water, and eating. These measurements allowed for the analysis of force exertion during different throat movements in male and female participants.
